# Assessing the use of anti-PD1 monotherapy as adjuvant therapy and determinants of treatment choice in stage III cutaneous melanoma in the US

**DOI:** 10.1186/s12885-024-12178-w

**Published:** 2024-03-27

**Authors:** Eric D. Whitman, Todor I. Totev, Shan Jiang, Wilson L. da Costa, Dmitri Grebennik, Hongjue Wang, Andra-Ecaterina Boca, Rajeev Ayyagari

**Affiliations:** 1 Atlantic Health System Cancer Care, Morristown, NJ USA; 2 Atlantic Melanoma Center, Morristown, NJ USA; 3 Analysis Group, Inc., Boston, MA USA; 4 Merck and Co., Inc., Rahway, NJ USA

**Keywords:** Adjuvant therapy, Stage III melanoma, Determinants of treatment choice

## Abstract

**Background:**

The objective of this study was to describe real-world adjuvant therapy (AT) use by disease substage and assess determinants of treatment choice among patients with stage III melanoma.

**Methods:**

This non-interventional retrospective study included survey responses and data from patient records provided by US medical oncologists. Survey responses, patient demographic/clinical characteristics, treatment utilization, and reasons for treatment were reported descriptively. The association between patient and disease characteristics and AT selection was assessed using logistic and multinomial regression models, overall and stratified by AJCC8 substage (IIIA vs. IIIB/C/D) and type of AT received (anti-PD1 monotherapy, BRAF/MEK, no AT), respectively.

**Results:**

In total 152 medical oncologists completed the survey and reviewed the charts of 507 patients (168 stage IIIA; 339 stages IIIB/IIIC/IIID); 405 (79.9%) patients received AT (360/405 (88.9%) received anti-PD1 therapy; 45/405 (11.1%) received BRAF/MEK therapy). Physicians reported clinical guidelines (61.2%), treatment efficacy (37.5%), and ECOG performance status (31.6%) as drivers of AT prescription. Patient-level data confirmed that improving patient outcomes (79%) was the main reason for anti-PD1 prescription; expected limited treatment benefit (37%), patient refusal (36%), and toxicity concerns (30%) were reasons for not prescribing AT. In multivariable analyses stage IIIB/IIIC/IIID disease significantly increased the probability of receiving AT (odds ratio [OR] 1.74) and anti-PD1 therapy (OR 1.82); ECOG 2/3 and Medicaid/no insurance decreased the probability of AT receipt (OR 0.37 and 0.42, respectively) and anti-PD1 therapy (OR 0.41 and 0.42, respectively) among all patients and patients with stage IIIA disease.

**Conclusion:**

Most patients were given AT with a vast majority treated with an anti-PD1 therapy. Physician- and patient-level evidence confirmed the impact of disease substage on AT use, with stage IIIA patients, patients without adequate insurance coverage, and worse ECOG status having a lower probability of receiving AT.

**Supplementary Information:**

The online version contains supplementary material available at 10.1186/s12885-024-12178-w.

## Introduction

Melanoma is the most severe form of skin cancer and can affect adults of all ages. The American Cancer Society estimates that about 97,610 people will be newly diagnosed with melanoma in the United States (US) in 2023, resulting in 7,990 deaths [1]. Surgical excision is the standard treatment for localized disease and regional lymph nodes (LNs), but patients with high-risk features in the primary tumor or regional LN metastasis have a high risk of recurrence [2]. Adjuvant cancer therapy is often recommended for these patients to reduce the risk of disease recurrence.

Immunotherapy with immune checkpoint inhibitors in the adjuvant setting has transformed the treatment of cutaneous melanoma. Pembrolizumab, an antibody against the programmed death 1 (PD1) receptor, was approved in February 2019 based on evidence from the KEYNOTE-054 trial [3, 4], in which it was associated with significantly longer progression-free survival and no new toxicities compared with placebo [4]. In a subsequent sub-analysis of the KEYNOTE-054 trial, the distant metastasis-free survival benefit favored pembrolizumab over placebo and was similar across American Joint Committee on Cancer 8th Edition (AJCC8) substages IIIA, IIIB, IIIC, and IIID [5]. Despite the promising clinical benefits associated with adjuvant immunotherapy for patients with stage III melanoma, some science leaders and clinical guidelines do not support the routine use of adjuvant therapy (AT) for AJCC8 [6] stage IIIA melanoma because of toxicity concerns and questions about the benefit of treatment in this population with better prognosis.

Given the recent advancement in adjuvant treatment, it is important to investigate adjuvant treatment patterns in a contemporary cohort of patients with stage III cutaneous melanoma in the US, as well as to better understand physician perceptions of AT in clinical practice and determinants of adjuvant treatment choice. In the present study, a survey of oncologists across different regions in the US was conducted along with a retrospective review of medical records of patients with complete resection of stage III cutaneous melanoma. The specific objectives of this study were to: 1) characterize treatment use, including the proportion of patients treated with anti-PD1 monotherapy and BRAF/MEK therapy; 2) describe patient and disease characteristics by adjuvant treatment choice (including no treatment) and AJCC 8th edition substage; and 3) assess the determinants of treatment choices (including no treatment) and physician perceptions of treatment drivers and unmet needs by AJCC 8th edition substage (e.g., IIIA vs. others).

## Methods

### Study design

This study was a non-interventional study including an online physician survey and a retrospective physician panel-based chart review using electronic medical records.

### Study participants

For physicians to be included in the survey, they had to have been a licensed medical oncologist in the US, have seen at least 5 adult patients with stage III melanoma in the past 5 years, have treated patients with melanoma in the adjuvant setting, be willing to consent, and not currently practice in the State of Vermont. A total of 325 physicians were contacted online via physician panel vendor. No physician identification data was available to the investigators. The physician panel was geographically dispersed in the US, and 152 physicians completed the survey.

For the retrospective chart review, medical oncologists from the nationwide panel identified patients with stage III cutaneous melanoma who had a surgical resection. Patients were included if they were ≥ 18 years old with a diagnosis of stage III cutaneous melanoma who had a surgical resection between January 1, 2018, and December 31, 2021, and whose charts had complete substage information and treatment data for at least 6 months before and 6 months after the index date were included for chart review. To avoid selection bias, physicians randomly selected the patients to be included using a predefined selection algorithm (Additional Methods, Additional File 1) in a 4:1 (AT / no AT proportion), with approximately 100 not treated patients. The 4:1 ratio was chosen to ensure that the group of patients who did not receive adjuvant therapy was not underrepresented, and sufficient sample size was available for the analyses. The *index date* was the date of surgical resection (wide local excision and sentinel LN [SLN] biopsy, or complete LN dissection [CLND]). The *pre-index period* was the period from initial melanoma diagnosis to the index date. The *post-index period* was the period from the index date to the end of follow-up or death, whichever occurred first (Supplemental Fig. 1, Additional File 1). Patients were excluded if they participated in a clinical trial before stage III cutaneous melanoma diagnosis, received neoadjuvant and/or adjuvant treatment for stage III melanoma in a clinical trial after diagnosis, or if they were diagnosed with an active second non-in-situ malignancy in the past 3 years prior to index date.

**Fig. 1:**
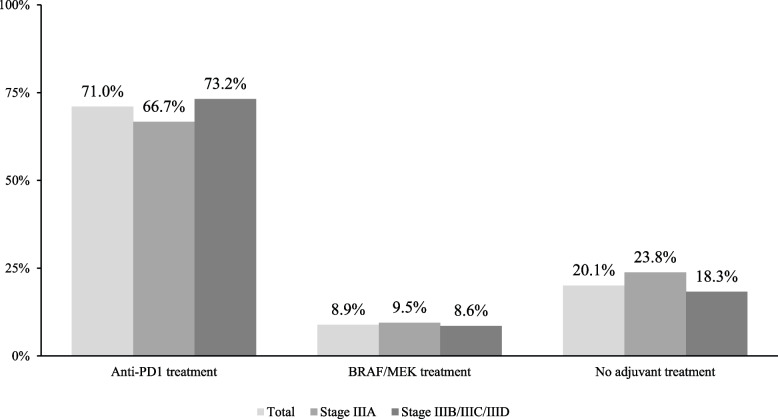
Adjuvant therapy use – overall and by disease substage

### Data collection

The online physician survey collected data on physician characteristics (i.e., specialty, practice setting, geographic region, years of medical practice, years of treating patients with cutaneous melanoma, number of stage III melanoma patients seen in the past 5 years, proportion of patients with stage III melanoma receiving adjuvant therapy, proportion of patients referred by dermatologists, surgical oncologists, primary care providers, or other specialties), and on physician perceptions about adjuvant treatment of patients with stage III melanoma (i.e., drivers of adjuvant treatment choice, characteristics of the high-risk patient population, treatment guideline referenced, frequency of shared decision making with patients regarding adjuvant therapy use, proportion of patients who refused adjuvant therapy, and reasons why patients refuse adjuvant therapy).

The retrospective chart review study collected information on demographics at resection (i.e., age, sex, race/ethnicity, geographic region, and insurance coverage), clinical characteristics (i.e., comorbidities, disease substage at diagnosis, Eastern Cooperative Oncology Group Performance Status [ECOG PS], pathology data, and laboratory tests, including lactate dehydrogenase [LDH] levels and BRAF mutation status), treatment history before resection, and surgical resection and treatment following resection (i.e., disease substage before resection, initial diagnosis or recurrence before resection, type of lymph node dissection, receipt of radiotherapy, and receipt of steroids after resection).

All data extracted from medical records were entered into the electronic case report form (eCRF). The physician survey was also integrated into the eCRF for data entry by participating physicians. In addition, automated logic and range checks were implemented within the eCRF to help ensure data quality at the point of entry. Before data collection, the eCRF was pretested with two eligible oncologists from the nationwide panel through one-on-one interviews to obtain input on clarity and ease of data entry. The eCRF and survey were reviewed by the physicians and revised based on feedback from these pretests. A “soft launch” of the eCRF and survey was then conducted with 54 patients. The quality of the soft launch data was reviewed manually; this included checking variable ranges and data consistency between related questions. Potential issues (e.g., areas of misinterpretation and data entry errors) were addressed before full launch of the eCRF and survey.

This study did not require patient informed consent, as only deidentified data were collected from the chart review. All study materials were reviewed by the WIRB-Copernicus Group Independent Review Board, which granted an exemption determination on November 29, 2022 per Title 45 of CFR, Part 46.104(d)(2, 4) as no personally identifiable information was collected [7].

### Statistical analysis

Descriptive analyses were used to report the data from the physician survey, patient demographics and clinical characteristics, and treatment utilization. Continuous variables were described using means, standard deviations, and medians; categorical variables were described using counts and percentages. Analyses were stratified by AJCC8 substage (IIIA vs. IIIB, IIIC, or IIID [IIIB/C/D]) and by adjuvant treatment received (i.e., anti-PD1 monotherapy, BRAF/MEK therapy, no treatment) and the subgroup that did not receive adjuvant treatment.

Reasons for AT selection (including no treatment) were also summarized by AJCC8 substage and adjuvant treatment received. Statistical comparisons between cohorts were performed using analysis of variance or the Wilcoxon rank-sum test for continuous variables and chi-squared test for categorical variables. For categorical variables with expected counts less than 10, Fisher’s exact test was used instead of the chi-squared test. Differences with *P*-values smaller than 0.05 were considered statistically significant.

The associations between patient and disease characteristics and AT use were assessed using logistic and multinomial regression models, overall and stratified by AJCC8 substage (IIIA vs. IIIB/C/D). In the logistic model, the dependent variable was whether a patient received AT; in the multinomial model, it was the type of AT received (anti-PD1 monotherapy, BRAF/MEK, or no AT). For both models, the independent variables were patient demographic and clinical characteristics including age, sex, insurance coverage, comorbidities on the index date, ECOG PS after the index date, baseline tumor ulceration, baseline LDH (above upper limit of normal, normal range, etc.), presence of lymphovascular invasion, presence of regression in primary specimen, BRAF mutation status after the index date, disease substage before resection on the index date, type of resection on the index date, receipt of radiotherapy after the index date, receipt of steroids after the index date, deep margin status, and year of surgical resection. Backward selection was used for both logistic and multinomial regressions to trim redundant predictors from the model; the final model chosen was the one with the lowest Akaike information criterion.

All analyses were conducted using RStudio Version 1.2.1335.

## Results

### Study sample

A total of 152 medical oncologists who had treated patients with stage III melanoma completed a survey based on their clinical experience and reviewed the charts of 507 patients diagnosed with stage III melanoma whom they had treated. Of these 507 patients, 405 (79.9%) received AT (360 received anti-PD1 therapy [222 pembrolizumab and 138 nivolumab] and 45 received BRAF/MEK therapy).

### Physician survey

Physicians most frequently practiced medicine in private practice (40.1%) or in an academic institution (38.2%) and lived in the South (37.5%) or Northeast (25.0%) region. They reported having treated patients with cutaneous melanoma for a mean of 15.3 years, half of them followed > 50 stage III melanoma patients in the past 5 years, and 75.8% of their stage III melanoma patients received AT after resection.

Physicians most frequently reported clinical guidelines (61.2%), treatment efficacy (37.5%), ECOG PS (31.6%), disease stage (21.7%), and patient comorbidities (18.4%) as the key drivers of AT prescription. Most physicians (76.7%) reported that over half of their patients had available BRAF mutation status information after surgery, and 68.0% usually or always considered BRAF mutation status when offering AT to their patients. In addition, the majority (55.9%) reported that the decision to use AT was usually or always shared with patients over half of of the time. A large proportion of physicians (59.9%) reported having had a patient refuse AT. The most commons reasons for patients refusing AT were concerns about treatment-related toxicity (68.1%), expected impact on daily life and daily activities (44.0%), cost/insurance coverage (37.4%), and comorbidities (e.g., autoimmune disease) (36.3%).

### Patient demographics and clinical characteristics from medical records

The mean age at the time of resection was 58.1 years, 56.0% of the patients were male, most patients were White (76.1%) and commercially insured (59.0%). At the time of resection, patients had substage IIIA (33.1%), IIIB (37.1%), or IIID (23.3%) disease (Table 1).

**Table 1:** Patient demographics and clinical characteristics

		**Substage category**			**Treatment category**			
	**Total**	**Stage IIIA**	**Stage IIIB/C/D**	***P*-value**	**Anti-PD1**	**BRAF/MEK**	**No AT**	***P*-value**
	***N* = 507**	***N* = 168**	***N* = 339**		***N* = 360**	***N* = 45**	***N* = 102**	
**Age at index date (years), mean ± SD [median]**	58.1 ± 11.8 [58.0]	58.8 ± 12.7 [61.0]	57.8 ± 11.3 [57.0]	0.42	58.0 ± 12.2 [57.5]	57.8 ± 11.6 [58.0]	58.7 ± 10.5 [59.0]	0.84
**Male sex, *n* (%)**	284 (56.0)	85 (50.6)	199 (58.7)	0.10	195 (54.2)	22 (48.9)	67 (65.7)	0.07
**Race, *n* (%)**^**a**^								
White	386 (76.1)	135 (80.4)	251 (74.0)	0.14	281 (78.1)	30 (66.7)	75 (73.5)	0.19
Black or African American	55 (10.8)	13 (7.7)	42 (12.4)	0.15	35 (9.7)	6 (13.3)	14 (13.7)	0.44
Asian	54 (10.7)	18 (10.7)	36 (10.6)	1.00	38 (10.6)	7 (15.6)	9 (8.8)	0.47
Other	13 (2.6)	2 (1.2)	11 (3.2)	0.24	7 (1.9)	2 (4.4)	4 (3.9)	0.27
**Hispanic ethnicity, *n* (%)**	54 (10.7)	11 (6.5)	43 (12.7)	0.05	31 (8.6)	8 (17.8)	15 (14.7)	0.06
**Insurance type, n (%)**^**a**^				0.28				< 0.01 *
Commercial/private insurance	299 (59.0)	98 (58.3)	201 (59.3)		299 (59.0)	220 (61.1)	31 (68.9)	
Medicare	131 (25.8)	48 (28.6)	83 (24.5)		131 (25.8)	97 (26.9)	10 (22.2)	
Medicaid/No insurance	67 (13.2)	17 (10.1)	50 (14.7)		67 (13.2)	37 (10.3)	4 (8.9)	
Other	10 (2.0)	5 (3.0)	5 (1.5)		10 (2.0)	6 (1.7)	0 (0.0)	
**Disease substage at resection date, *n* (%)**				< 0.001 *				< 0.01 *
IIIA	168 (33.1)	168 (100.0)	0 (0.0)		112 (31.1)	16 (35.6)	40 (39.2)	
IIIB	188 (37.1)	0 (0.0)	188 (55.5)		133 (36.9)	11 (24.4)	44 (43.1)	
IIIC	118 (23.3)	0 (0.0)	118 (34.8)		92 (25.6)	10 (22.2)	16 (15.7)	
IIID	33 (6.5)	0 (0.0)	33 (9.7)		23 (6.4)	8 (17.8)	2 (2.0)	
**Main comorbidities at resection date, *n* (%)**^**a**^								
Any comorbidity	363 (71.6)	117 (69.6)	246 (72.6)	0.56	256 (71.1)	32 (71.1)	75 (73.5)	0.89
Hypertension	196 (38.7)	60 (35.7)	136 (40.1)	0.39	135 (37.5)	16 (35.6)	45 (44.1)	0.43
Coronary artery disease/myocardial infarction	60 (11.8)	12 (7.1)	48 (14.2)	< 0.05 *	31 (8.6)	7 (15.6)	22 (21.6)	< 0.01 *
Asthma	48 (9.5)	17 (10.1)	31 (9.1)	0.85	35 (9.7)	7 (15.6)	6 (5.9)	0.17
Anxiety disorder	41 (8.1)	15 (8.9)	26 (7.7)	0.75	29 (8.1)	4 (8.9)	8 (7.8)	0.96
Depression	38 (7.5)	11 (6.5)	27 (8.0)	0.70	31 (8.6)	1 (2.2)	6 (5.9)	0.32
**Performance status after resection, *n* (%)**								
ECOG-PS after resection				< 0.01 *				< 0.001 *
0	167 (32.9)	66 (39.3)	101 (29.8)		135 (37.5)	9 (20.0)	23 (22.5)	
1	249 (49.1)	70 (41.7)	179 (52.8)		176 (48.9)	32 (71.1)	41 (40.2)	
2	61 (12.0)	16 (9.5)	45 (13.3)		32 (8.9)	3 (6.7)	26 (25.5)	
3	9 (1.8)	2 (1.2)	7 (2.1)		4 (1.1)	0 (0.0)	5 (4.9)	
Unknown/not sure	21 (4.1)	14 (8.3)	7 (2.1)		13 (3.6)	1 (2.2)	7 (6.9)	
**Baseline LDH level on resection date or in the preceding 6 months (*N* = 370), *n* (%)**				< 0.001 *				0.45
Normal range	231 (62.4)	96 (78.7)	135 (54.4)		172 (64.4)	20 (57.1)	39 (57.4)	
Above upper limit of normal	139 (37.6)	26 (21.3)	113 (45.6)		95 (35.6)	15 (42.9)	29 (42.6)	
**BRAF mutated/positive, *n* (%)**	157 (31.0)	59 (35.1)	98 (28.9)	0.19	90 (25.0)	36 (80.0)	31 (30.4)	< 0.001 *
**Pathological characteristics, *n* (%)**								
Ulceration	268 (52.9)	75 (44.6)	193 (56.9)	< 0.05 *	195 (54.2)	26 (57.8)	47 (46.1)	0.51
Deep margin status				< 0.001 *				0.48
Positive	113 (22.3)	24 (14.3)	89 (26.3)		76 (21.1)	11 (24.4)	26 (25.5)	
Negative	368 (72.6)	139 (82.7)	229 (67.6)		267 (74.2)	30 (66.7)	71 (69.6)	
Transected	19 (3.7)	2 (1.2)	17 (5.0)		12 (3.3)	4 (8.9)	3 (2.9)	
Unknown/not reported	7 (1.4)	3 (1.8)	4 (1.2)		5 (1.4)	0 (0.0)	2 (2.0)	
Lymphovascular/ angiolymphatic invasion	211 (41.6)	56 (33.3)	155 (45.7)	< 0.05 *	152 (42.2)	21 (46.7)	38 (37.3)	0.47

*Abbreviations: AT* adjuvant therapy, *ECOG-PS* Easton Cooperative Oncology Group Performance Status, *LDH* lactate dehydrogenase, *SD* standard deviation

^a^As multiple responses were allowed for this question, percentages may add up to more than 100%

*P-value < 0.05

A total of 71.6% of patients had at least one comorbidity, and 82.0% had an ECOG PS of 0 or 1. The most common comorbidities were hypertension (38.7%), coronary artery disease/myocardial infarction (11.8%), asthma (9.5%), anxiety disorder (8.1%), and depression (7.5%; Table 1). Baseline LDH levels on the date of resection or in the preceding 6 months were obtained for 370 patients. Of these, 231 (62.4%) had normal LDH levels and 139 (37.6%) had levels that were above the upper limit of normal. In addition, BRAF mutation was found in 31.0% of the patients (Table 1). Pathological findings included ulcerations, observed in 52.9% of patients, lymphovascular invasion in 41.6%, and positive deep margins in 22.3% (Table 1).

### Treatment utilization

A total of 81.6% of patients had the primary lesion resected before the main surgical resection. Most patients (92.5%) had a resection following initial melanoma diagnosis, while 6.9% had a resection following disease recurrence. The resection included a complete lymph node dissection among 74.6% of the patients, while 18.9% had sentinel node biopsy only (Supplemental Table 1, Additional File 1). After resection, 26.0% of patients received radiotherapy, either in the primary site (80.3%) or in the lymph node basin (68.9%). About 10.5% of patients received steroids after surgical resection (Supplemental Table 1, Additional File 1).

Among the 507 patients in the study, 71.0% received AT with anti-PD1, 8.9% had BRAF/MEK therapy, and 20.1% did not receive any AT (Fig. 1). Further, patients resected in recent years were significantly more likely to receive anti-PD1 therapy after resection compared to BRAF/MEK or no treatment (Supplemental Fig. 2, Additional File 1). Among patients treated with adjuvant anti-PD1 therapy, 222 (54.8%) received pembrolizumab and 138 (34.1%) received nivolumab (Table 2). The mean time from resection to treatment initiation was 37 days. Median duration of AT was 357 days, 78.4% of patients discontinued treatment, and 25 (6.2%) had a change in dosage or dose frequency (Table 2).

**Table 2:** Adjuvant therapy after surgical resection

		**Substage category**			**Treatment category**		
	**Total**	**Stage IIIA**	**Stage IIIB/C/D**	***P*-value**	**Anti-PD1**	**BRAF/MEK**	***P*-value**
	***N* = 507**	***N* = 168**	***N* = 339**		***N* = 360**	***N* = 45**	
**Adjuvant therapy, *n* (%)**							-
Pembrolizumab	222 (54.8)	62 (48.4)	160 (57.8)		222 (61.7)	0 (0.0)	-
Nivolumab	138 (34.1)	50 (39.1)	88 (31.8)		138 (38.3)	0 (0.0)	-
Dabrafenib	45 (11.1)	16 (12.5)	29 (10.5)		0 (0.0)	45 (100.0)	-
Trametinib	45 (11.1)	16 (12.5)	29 (10.5)		0 (0.0)	45 (100.0)	0.41
**Time from resection to treatment initiation (days) (N = 350)**^**a**^**, mean ± SD [median]**	36.9 ± 32.5 [30.0]	33.1 ± 27.0 [28.0]	38.6 ± 34.6 [30.0]	0.15	37.7 ± 33.9 [30.0]	29.7 ± 16.3 [27.0]	1.00
** Patients who discontinued treatment (*N* = 329), *n* (%)**^**a**^	258 (78.4)	82 (80.4)	176 (77.5)	0.66	231 (78.3)	27 (79.4)	0.98
Duration of AT (days) (*N* = 258)^a^, mean ± SD [median]	327.8 ± 138.5 [357.0]	332.8 ± 141.0 [357.0]	325.6 ± 137.6 [355.0]	0.58	328.0 ± 141.6 [357.0]	326.1 ± 110.5 [357.0]	1.00
** Patients with changes in adjuvant treatment, *n* (%)**	25 (6.2)	5 (3.9)	20 (7.2)	0.29	23 (6.4)	2 (4.4)	

*Abbreviations:* *AT* adjuvant therapy, *SD* standard deviation

^a^Total number of patients with available information

### Reasons for AT prescription

**Fig. 2:**
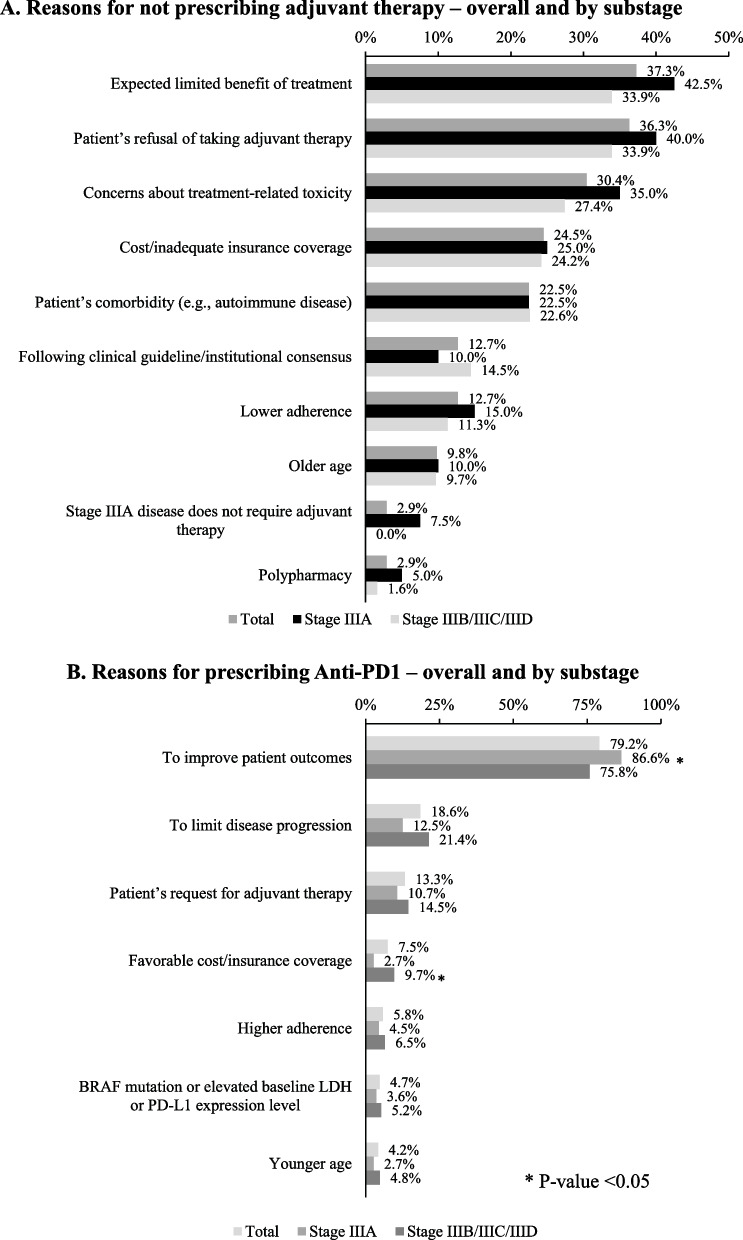
Reasons for adjuvant therapy selection. **a** Reasons for not prescribing adjuvant therapy – overall and by substage. **b** Reasons for prescribing Anti-PD1 – overall and by substage. * *P*-value < 0.05. Abbreviations: LDH, lactate dehydrogenase

The primary documented reasons in the patient charts for not prescribing AT included expected limited benefits of treatment (37.3%), patient’s refusal of AT (36.3%), concerns about treatment-related toxicity (30.4%), and cost/inadequate insurance coverage (25%; Fig. 2A). The documented main reasons for physicians selecting anti-PD1 therapy were to improve patient outcomes (79.2%), to limit disease progression (18.6%), patient request (13.3%), favorable cost/insurance coverage (7.5%), and high adherence (5.8%; Fig. 2B).

### Determinants of treatment choices

**Fig. 3:**
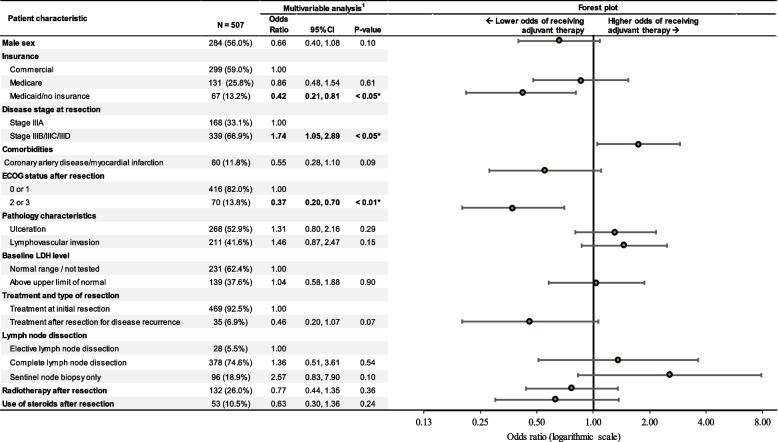
Determinants of adjuvant treatment choice among all patients with stage III melanoma. Abbreviations: CI, confidence interval; ECOG, Eastern Cooperative Oncology Group; LDH, lactate dehydrogenase

Among all stage III patients, compared with patients who did not receive AT, having disease substages IIIB/C/D significantly increased the probability of receiving AT (odds ratio [OR] = 1.74; 95% confidence interval [CI]: 1.05, 2.89; *P* < 0.05). Additionally, Medicaid/no insurance (OR = 0.42, 95% CI: 0.21, 0.81; *P* < 0.05) and ECOG PS of 2/3 (OR = 0.37, 95% CI: 0.20, 0.70; *P* < 0.01) were associated with a significantly lower probability of receiving AT (Fig. 3).

### Subgroup analysis by substage

The mean age at the time of resection was 58.8 years for patients with stage IIIA disease and 57.8 years for patients with stage IIIB/C/D disease; 50.6% among stage IIIA patients and 58.7% among stage IIIB/C/D patients were male (Table 1).

The presence of comorbidities was similar between stage subgroups (69.6% substage IIIA and 72.6% substages IIIB/C/D). Patients with coronary artery disease/myocardial infraction were more frequent in the IIIB/C/D substage (14.2% vs. 7.1% in the stage IIIA subgroup). The proportion of patients with an ECOG PS of 0 was higher in the IIIA subgroup (39.3% vs. 29.8% in the IIIB/C/D subgroup), whereas the proportion with an ECOG PS of 1 was higher in the IIIB/C/D subgroup (52.8% vs. 41.7%; both *P* < 0.01; Table 1). A significantly higher proportion of patients in the IIIB/C/D subgroup had elevated levels of LDH compared to patients in the IIIA subgroup (45.6% vs. 21.3%)**.** Patients in the IIIB/C/D substages had significantly higher proportions of ulceration, positive margin status, and lymphovascular invasion compared to patients in the IIIA substage (Table 1). Patients with stage IIIA more frequently had a resection of the primary lesion before the main surgical procedure (91.4% vs. 77.3%). No other significant differences were observed in treatment utilization between substages (Supplemental Table 1, Additional File 1).

Improving patient outcomes was a significantly more common reason for selecting anti-PD1 therapy in the stage IIIA subgroup (86.6%) compared to the IIIB/C/D subgroup (75.8%; Fig. 2B). Meanwhile, favorable cost/insurance coverage was a more common factor for anti-PD1 therapy selection among patients in the IIIB/C/D subgroup (9.7%) compared to patients in the IIIA subgroup (2.7%; Fig. 2B).

The multivariable regression analysis identified several factors associated with adjuvant treatment choice stratified by disease substage. For patients with stage IIIA disease, SLN biopsy (OR = 23.63, 95% CI: 3.29, 169.94; *P* < 0.01) and CLND (OR = 5.52, 95% CI: 1.10, 27.70; *P* < 0.05 vs. elective dissection), and baseline LDH levels above the upper limit of normal (OR = 4.46, 95% CI: 1.06, 18.75; P < 0.05) were associated with a significantly higher probability of receiving AT. Medicaid/no insurance status (OR = 0.13, 95% CI: 0.04, 0.42; *P* < 0.001), ECOG PS of 2/3 (OR = 0.27, 95% CI: 0.08, 0.90; *P* < 0.05), and receipt of steroids (OR = 0.23, 95% CI: 0.06, 0.90; *P* < 0.05) were associated with a significantly lower probability of receiving AT (Supplemental Fig. 3, Additional File 1). For patients with stage IIIB/C/D disease, ECOG PS of 2/3 (OR = 0.28, 95% CI: 0.14, 0.57; *P* < 0.001), treatment after resection for disease recurrence (OR = 0.38, 95% CI: 0.14, 0.98; *P* < 0.05 vs. treatment at initial resection), and receipt of radiotherapy after resection (OR = 0.50, 95% CI: 0.27, 0.93; *P* < 0.05) were associated with a significantly lower probability of receiving AT (Supplemental Fig. 4, Additional File 1).

### Subgroup analysis by treatment category

Patients in each treatment group had mean age of approximately 58 years, and the proportion of males was numerically higher among not treated patients (65.7% vs. 54.2% in the anti-PD1 subgroup, and 48.9% in the BRAF/MEK subgroup). The subgroup of not treated patients had a significantly lower proportion of commercially insured patients and higher proportion of Medicaid patients compared to the anti-PD1 and BRAF/MEK subgroups. About 17.7% of patients in the not treated group had stage IIIB/C/D disease compared with 32.0% in the anti-PD1 therapy subgroup and 40.0% in the BRAF/MEK therapy subgroup (*P* < 0.01; Table 1). Patients in the not treated group were significantly more likely to have higher ECOG PS (e.g., 30.4% of these patients had an ECOG PS of 2/3 compared with 10.0% in the anti-PD1 therapy subgroup and 6.7% in the BRAF/MEK therapy subgroup; *P* < 0.001; Table 1).

Regarding treatment utilization, the proportion of patients who received radiotherapy after resection was significantly higher among not treated patients (37.3%) compared to patients who received anti-PD1 (22.2%) or BRAF/MEK therapy (31.1%).

The multinomial logistic regression model identified several factors associated with AT choice (anti-PD1 or BRAF/MEK therapy vs. no AT; Table 3). Compared with patients who did not receive AT, stage IIIB/C/D disease was associated with a significantly higher probability of receiving treatment with anti-PD1 therapy (OR = 1.82, 95% CI: 1.09, 3.05; *P* < 0.05). Additionally, Medicaid/no insurance status (OR = 0.43, 95% CI: 0.22, 0.84; *P* < 0.05), ECOG PS of 2/3 (OR = 0.41, 95% CI: 0.22, 0.79; *P* < 0.01), and history of coronary artery disease/myocardial infarction (OR = 0.49, 95% CI: 0.24, 0.98; *P* < 0.05) were associated with a significantly lower probability of receiving anti-PD1 therapy. ECOG PS of 2/3 (OR = 0.16, 95% CI: 0.04, 0.63; *P* < 0.01) was also associated with a significantly lower probability of receiving adjuvant BRAF/MEK therapy.

**Table 3:** Determinants of adjuvant treatment choice stratified by AT type

Patient characteristic	Total (*N* = 507)	Treatment category					
		**Anti-PD1 (*N* = 360)**			**BRAF/MEK (*N* = 45)**		
		**OR**	**95% CI**	***P*-value**	**OR**	**95% CI**	***P*-value**
**Male sex, *n* (%)**	284 (56.0)	0.68	(0.41, 1.13)	0.13	0.51	(0.24, 1.09)	0.08
**Insurance coverage, *n* (%)**							
Commercial	301 (59.4)	1.00			1.00		
Medicare	173 (34.1)	0.90	(0.49, 1.62)	0.72	0.63	(0.25, 1.55)	0.31
Medicaid/no insurance	67 (13.2)	0.43	(0.22, 0.84)	< 0.05 *	0.34	(0.10, 1.15)	0.08
**Disease substage at resection, *n* (%)**							
Stage IIIA	168 (33.1)	1.00					
Stage IIIB/C/D	339 (66.9)	1.82	(1.09, 3.05)	< 0.05 *	1.22	(0.55, 2.69)	0.63
**ECOG-PS after resection, *n* (%)**							
ECOG-PS 0/1	416 (82.0)	1.00					
ECOG-PS 2/3	70 (13.8)	0.41	(0.22, 0.79)	< 0.01 *	0.16	(0.04, 0.63)	< 0.01 *
**Comorbidities, *n* (%)**							
Coronary artery disease/myocardial infarction	41 (8.1)	0.49	(0.24, 0.98)	< 0.05 *	1.36	(0.47, 3.94)	0.58
**Pathology characteristics, *n* (%)**							
Ulceration	268 (52.9)	1.31	(0.79, 2.16)	0.30	1.38	(0.63, 3.00)	0.42
Lymphovascular invasion	211 (41.6)	1.45	(0.85, 2.46)	0.17	1.58	(0.71, 3.52)	0.26
**Baseline LDH level, *n* (%)**							
Normal range/not tested	368 (72.6)	1.00					
Above upper limit of normal	139 (27.4)	1.01	(0.55, 1.84)	0.98	1.35	(0.56, 3.24)	0.51
**Treatment and type of resection, *n* (%)**							
Treatment at initial resection	469 (92.5)	1.00					
Treatment after resection for disease recurrence	35 (6.9)	0.44	(0.19, 1.05)	0.06	0.63	(0.15, 2.58)	0.52
**LN dissection, *n* (%)**							
Elective LN dissection	28 (5.5)	1.00					
Complete LN dissection	378 (74.6)	1.33	(0.50, 3.58)	0.57	1.62	(0.30, 8.85)	0.57
SLN biopsy only	96 (18.9)	2.53	(0.81, 7.85)	0.11	2.63	(0.39, 17.76)	0.32
**Radiotherapy after resection, *n* (%)**	132 (26.0)	0.75	(0.43, 1.33)	0.32	0.96	(0.40, 2.27)	0.92
**Use of steroids after resection, *n* (%)**	53 (10.5)	0.56	(0.25, 1.22)	0.14	1.32	(0.44, 3.93)	0.62

*Abbreviations: CI* confidence interval, *ECOG-PS* Eastern Cooperative Oncology Group, *LDH* lactate dehydrogenase, *LN* lymph nodes, *OR* odds ratio

*P-value < 0.05

## Discussion

The use of AT for stage III melanoma is debated for patients with low risk of recurrence, including patients with AJCC8 IIIA [6] and SLN metastasis with < 1 mm of tumor depth [8–10]. Clinical guidelines for stage IIIA patients—who constituted a very small fraction of patients included in the pivotal clinical trials leading to the approval of nivolumab, pembrolizumab, and dabrafenib/trametinib [4, 11–14]—are not well-defined. As the adjuvant treatment landscape continues to expand, it is important to determine the extent of adjuvant treatment use, particularly if patients with stage IIIA have been undertreated, in real-world practice and motivations thereof to ensure that patients are receiving adequate and appropriate care.

To address this question, the present study examined adjuvant treatment patterns in patients with stage III melanoma through a cross-sectional survey of oncologists from different regions in the US and retrospective review of medical records, spanning a period after the introduction of AJCC8 staging [6] and the approval of newer adjuvant therapies (January 2018 through December 2021). In this study, anti-PD1 therapy was offered to the vast majority (88.8%) of patients who received AT, including among patients with BRAF mutations (71.4%). Patients in the stage IIIB/C/D subgroup had less favorable clinical characteristics than patients with stage IIIA, namely a higher rate of coronary artery disease and higher levels of LDH, but also had a higher probability of receiving AT compared to patients with stage IIIA.

The physician survey revealed that the main factor motivating the prescription of AT to patients with stage III melanoma was clinical guidelines. This is similar to the findings of a multi-country chart review study, which reported that in 88% of cases, physicians elected to initiate AT because it was an approved treatment option [15]. The physician survey also showed that treatment efficacy and ECOG PS (which was 0 or 1 in > 80% of patients after resection) were important considerations for the use of AT. Treatment guidelines for adjuvant treatment of AJCC8 stage IIIA SLN-positive melanoma could be clearer as well. The American Society of Clinical Oncology [16] and National Comprehensive Cancer Network [17] recommend systemic therapy with an anti-PD1 antibody (nivolumab or pembrolizumab) or, for patients with BRAF V600 activating mutation, the combination of dabrafenib and trametinib, with the caveat that for stage IIIA with low risk of recurrence, the toxicity of these treatments should be weighed against the benefits. While concerns about treatment-related toxicity was the third main reason for physicians not prescribing AT according to results from the survey, it was the main reason for patient treatment refusal. Furthermore, just over half of physicians reported that the decision to use AT was usually or always shared with patients more than half of the time. This suggests that improving patient education about the risks/benefits of AT as well as improving shared decision making between the physician and their patient could lead to better treatment choices and adherence.

The findings from the present investigation are corroborated by other studies. In a survey of physicians and nurses treating patients with stage III melanoma at three melanoma centers in Australia (2019–2020), the major factors guiding the decision on whether to initiate adjuvant treatment were disease substage (IIIC/D vs. IIIA/B) and patients’ age (> 80 vs. ≤ 80 years) and ECOG PS; secondary considerations were treatment effectiveness and tolerability to patients [18]. In a systematic review of eight studies that examined factors influencing patients’ and physicians’ decision to initiate adjuvant immunotherapy, survival was the principal concern, superseding tolerability; others were relapse-free survival, factors related to the drug regimen, treatment costs, and quality of life [19]. In contrast, the multi-country chart review study found that concern over treatment toxicity was the main reason for which surveillance was preferred over AT [15].

We performed multivariable analysis stratified by disease substage and treatment to identify factors associated with AT receipt. Among patients with stage IIIA disease, undergoing SLN biopsy only, undergoing CLND, and elevated baseline LDH levels were associated with an increased probability of AT receipt, whereas Medicaid/no insurance status, ECOG PS of 2 or 3, and receipt of steroids were associated with a decreased probability of AT receipt. These results suggest that oncologists more frequently offer AT for stage IIIA patients who have characteristics that are suggestive of higher risk disease, better performance status, and/or fewer comorbidities.

Among patients with stage IIIB/C/D disease, ECOG PS of 2 or 3, treatment after resection for disease recurrence (vs. at initial resection), and receipt of radiotherapy were associated with a reduced probability of receiving AT. Disease substage has previously been shown to be related to receipt of adjuvant treatment; in the NCDB study (2004–2015), there was a correlation between disease substage and use of any AT (IIIA, 33%; IIIB, 39%; IIIC, 52%) [20]. Additionally, in a large-scale German study conducted at multiple skin cancer centers, a larger proportion of patients with stage IIID disease (89%) and a smaller proportion with stage IIIA (72%) elected to receive adjuvant treatment [21]. In the subgroup analysis by treatment, patients who did not receive AT were more frequently covered by Medicaid/had no insurance, had stage IIIA disease at diagnosis, were more likely to have worse ECOG PS, and to receive radiotherapy after resection. The lower probability of AT receipt among patients with stage IIIA disease might be influenced not only by clinical factors, but also by oncologists’ interpretation of clinical guidelines and the discussions that follow that might lead patients to refuse taking AT.

There have been no direct comparisons of targeted therapy vs. immunotherapy and therefore, there are no data to support the use of one or the other as the first adjuvant treatment option. In this study, approximately one-third of patients harbored BRAF mutation, but patients were far more likely to receive anti-PD1 therapy than BRAF/MEK inhibitor or no treatment following resection, even among patients with BRAF mutation. The review of patient charts showed that the main reasons for choosing adjuvant anti-PD1 therapy were to limit disease progression and cost/insurance considerations. In contrast, a previous study found that similar proportions of patients chose checkpoint inhibitor and targeted therapy (53% and 47%, respectively) among patients with BRAF mutation who initiated adjuvant treatment [21].

Patients in the current study who received adjuvant anti-PD1 therapy were less frequently treated before resection, less frequently received radiotherapy or steroids after resection, and were more likely to receive the standard dose of AT for the recommended 1-year period. The choice of adjuvant treatment is typically made based on the patient’s performance status, comorbidities, and age [22], and the benefits of a particular treatment must be weighed against the risks. For example, higher rates of grade 3 or 4 AEs have been reported with the dabrafenib and trametinib combination than with anti-PD1 therapies [22], but the latter are associated with long-term endocrine complications [23].

Most patients in this study had CLND as part of their surgical procedures. However, two randomized studies published in 2016 and 2017 found no additional survival benefit with CLND after positive SLN dissection [24, 25], and it is no longer included in the treatment algorithm for stage III melanoma [17]. The high rate of CLNDs in the current study may reflect a persistence of this practice—especially at smaller clinics—during the study period.

Strengths of this chart review study include the substantial number of patients with stage III melanoma from various practice settings across the US providing valuable real-world evidence of the management of resected melanoma in the US. Additionally, substage III data were used to identify factors associated with AT receipt among patients with stage IIIA disease and the drivers behind these choices; this had not been explored in previous real-world studies [21, 26]. This study also used extensive information from different sources – a physician survey to describe their clinical practice experience and patient-level data obtained from chart review.

Findings from this study are subject to limitations. First, as a retrospective observational study, the identification of patients, disease characteristics, and treatments was limited to the availability and accuracy of patient charts and data collection procedure. Since data in patients’ medical charts may not have been collected for research purposes, detailed information was not available for all the study variables. In addition, the level of detail recorded varied across patients. Second, prescription and dispensing information available in the database were collected with the assumption that the medication was administered as prescribed. Third, misspecification or inaccuracies in the data collection process may result in misclassification of patient characteristics or of endpoints of interest. Quality assurance and data management practices discussed were applied to mitigate potential biases. Finally, patient reported outcomes data was not collected, thus excluding the patients’ experiences and perspective with AT.

## Conclusions

In a real-world setting, physicians chose AT for patients with melanoma based on clinical guidelines, cancer stage, and risk/benefit assessment, while lack of adequate insurance coverage and ECOG PS were associated with not using AT. Physicians also reported sharing the decision-making on the use of AT with approximately half of their patients, suggesting room for improvement with regards to patient education on the risks and benefits of AT initiation. Patient-level evidence identified the expected limited benefit of AT and patient refusal as the primary reasons for not prescribing AT, particularly among patients with stage IIIA disease, who received AT less frequently, while improving patient outcomes and limiting disease progression were pointed out as the mean reasons for PD-1 prescription. In addition, stage IIIA disease, insurance status, higher ECOG PS, and history of coronary artery disease/myocardial infarction were confirmed as factors that hinder the prescription of AT among patients with melanoma. AT was offered to the majority of patients. Furthermore, anti-PD1 therapy was offered to the vast majority of patients who received AT, even among patients with BRAF mutations. These findings contribute to a better understanding of the current treatment patterns among patients with stage III cutaneous melanoma and the drivers to inform the development of new treatment.

### Supplementary Information

**Additional file 1:** **Supplemental Figure 1.** Study design for retrospective chart review. **Supplemental Figure 2.** Year of surgical resection – by adjuvant therapy. **Supplemental Figure 3.** Determinants of adjuvant treatment choice among patients with stage IIIA melanoma. Abbreviations: CI, confidence interval; ECOG, Eastern Cooperative Oncology Group; LDH, lactate dehydrogenase. **Supplemental Figure 4.** Determinants of adjuvant treatment choice among patients with stage IIIB/IIIC/IIID melanoma. Abbreviations: CI, confidence interval; ECOG, Eastern Cooperative Oncology Group.

## Data Availability

The data that support the findings of this study are available from Analysis Group, but restrictions apply to the availability of these data, which were used under license for the current study, and so are not publicly available. Data are however available from the authors upon reasonable request to the corresponding author and with permission of Merck & Co., Inc., Rahway, NJ, USA.

## Abbreviations

AJCC8: American Joint Committee on Cancer 8th edition
AT: Adjuvant therapy
CI: Confidence interval
CLND: Complete lymph node dissection
eCRF: Electronic case report form
ECOG PS: Eastern Cooperative Oncology Group Performance Status
LDH: Lactate dehydrogenase
LN: Lymph nodes
OR: Odds ratio
PD1: Programmed death 1 receptor
SLN: Sentinel lymph node
US: United States of America
