# Supplementary material for: Assessing the use of anti-PD1 monotherapy as adjuvant therapy and determinants of treatment choice in stage III cutaneous melanoma in the US
Source: BMC Cancer. 2024 Mar 27;24:389. doi: 10.1186/s12885-024-12178-w (PMC10967219; doi:10.1186/s12885-024-12178-w)

**Assessing the use of anti-PD1 monotherapy as adjuvant therapy and determinants of treatment choice in stage III cutaneous melanoma in the US**

Additional File 1

**Additional Methods**

Participating physicians from the nationwide panel identified and selected eligible patients from their practice using the below pre-defined algorithm and facilitated by an electronic case report form.

1. The physician was presented with a random letter of the alphabet and instructed to search their patient records to identify all eligible patients whose last name began with that letter. Eligible patients were required to meet the inclusion/exclusion criteria outlined in the *Study participants* sub-section of the *Methods* section in the manuscript.

- If there were no such patients, the physician was presented with another random letter.
- If the physician identified a patient, the patient’s data were extracted for the study.
- If the physician identified more than one patient, a single patient who was first in the list when the patients were ordered alphabetically by last name was selected for data extraction.

1. After the physician completed data entry, they were returned to Step 1—i.e., presented with another random letter to repeat the process for selecting the next patient.

This process was repeated until the physician extracted data from up to 5 patient medical records.

**Supplemental** **Table 1. Treatment utilization**

|  |  |  | **Substage category** | | |  | **Treatment category** | | | |
| --- | --- | --- | --- | --- | --- | --- | --- | --- | --- | --- |
|  | **Total** |  | **Stage IIIA** | **Stage IIIB/C/D** | **P-value** |  | **Anti-PD1** | **BRAF/MEK** | **No AT** | **P-value** |
|  | **N = 507** |  | **N = 168** | **N = 339** |  |  | **N = 360** | **N = 45** | **N = 102** |  |
| **Resection of the primary lesion before the main surgical procedure^1^** | 252 (81.6%) |  | 85 (91.4%) | 167 (77.3%) | <0.01* |  | 172 (85.1%) | 25 (80.6%) | 55 (72.4%) | < 0.05* |
| **Timing of surgical resection** |  |  |  |  | 0.51 |  |  |  |  | 0.09 |
| At initial melanoma diagnosis | 469 (92.5%) |  | 155 (92.3%) | 314 (92.6%) |  |  | 339 (94.2%) | 42 (93.3%) | 88 (86.3%) |  |
| After disease recurrence | 35 (6.9%) |  | 11 (6.5%) | 24 (7.1%) |  |  | 19 (5.3%) | 3 (6.7%) | 13 (12.7%) |  |
| Unknown/not sure | 3 (0.6%) |  | 2 (1.2%) | 1 (0.3%) |  |  | 2 (0.6%) | 0 (0.0%) | 1 (1.0%) |  |
| **Type of lymph node dissection on index date** |  |  |  |  | 0.23 |  |  |  |  | 0.61 |
| Complete LN dissection | 378 (74.6%) |  | 119 (70.8%) | 259 (76.4%) |  |  | 260 (72.2%) | 36 (80.0%) | 82 (80.4%) |  |
| SLN biopsy only | 96 (18.9%) |  | 38 (22.6%) | 58 (17.1%) |  |  | 75 (20.8%) | 7 (15.6%) | 14 (13.7%) |  |
| Elective LN dissection | 28 (5.5%) |  | 8 (4.8%) | 20 (5.9%) |  |  | 20 (5.6%) | 2 (4.4%) | 6 (5.9%) |  |
| Unknown | 5 (1.0%) |  | 3 (1.8%) | 2 (0.6%) |  |  | 5 (1.4%) | 0 (0.0%) | 0 (0.0%) |  |
| **Receipt of radiotherapy after resection** |  |  |  |  | 0.56 |  |  |  |  | < 0.05* |
| Yes | 132 (26.0%) |  | 39 (23.2%) | 93 (27.4%) |  |  | 80 (22.2%) | 14 (31.1%) | 38 (37.3%) |  |
| No | 369 (72.8%) |  | 127 (75.6%) | 242 (71.4%) |  |  | 275 (76.4%) | 30 (66.7%) | 64 (62.7%) |  |
| Unknown/not sure | 6 (1.2%) |  | 2 (1.2%) | 4 (1.2%) |  |  | 5 (1.4%) | 1 (2.2%) | 0 (0.0%) |  |
| **Site of radiotherapy^2,3^** |  |  |  |  |  |  |  |  |  |  |
| Primary site | 106 (80.3%) |  | 32 (82.1%) | 74 (79.6%) | 0.93 |  | 66 (82.5%) | 11 (78.6%) | 29 (76.3%) | 0.72 |
| LN basin | 91 (68.9%) |  | 24 (61.5%) | 67 (72.0%) | 0.33 |  | 59 (73.8%) | 9 (64.3%) | 23 (60.5%) | 0.32 |
| **Receipt of steroids after resection** |  |  |  |  | 0.24 |  |  |  |  | < 0.05* |
| Yes | 53 (10.5%) |  | 13 (7.7%) | 40 (11.8%) |  |  | 29 (8.1%) | 8 (17.8%) | 16 (15.7%) |  |
| No | 441 (87.0%) |  | 149 (88.7%) | 292 (86.1%) |  |  | 322 (89.4%) | 34 (75.6%) | 85 (83.3%) |  |
| Unknown/not sure | 13 (2.6%) |  | 6 (3.6%) | 7 (2.1%) |  |  | 9 (2.5%) | 3 (6.7%) | 1 (1.0%) |  |

| **Notes:** |  |  |  |  |  |  |  |  |  |  |
| --- | --- | --- | --- | --- | --- | --- | --- | --- | --- | --- |
| [1] Patients who did not receive any treatment or undergo resection of the primary lesion before the main surgical resection had likely undergone a biopsy. | | | | | | | | | | |
| [2] Percentages refer to the number of patients who received radiotherapy | | | | | | | | | | |
| [3] As multiple responses were allowed for this question, percentages may add up to more than 100%. | | | | | | | | | | |
| **Abbreviations:** AT, adjuvant therapy; LN, lymph nodes. | | | | | | | | | | |

**Supplemental Figure 1. Study design for retrospective chart review**

**Index date:**

**Date of surgical resection**

Initial melanoma diagnosis

End of follow-up or death

**Pre-index period**

- Diagnosis information
- Comorbidities
- Specialties of diagnosing and referring physicians
- Laboratory tests
- Pre-resection treatment history

**Post-index period**

- - Treatments following resection

**On index date**

- Demographic characteristics
- Disease substage
- ECOG-PS score
- Tumor pathology
- Surgical resection characteristics

**Supplemental Figure 2. Year of surgical resection – by adjuvant therapy**

**Supplemental Figure 3. Determinants of adjuvant treatment choice among patients with stage IIIA melanoma**

**Supplemental Figure 4. Determinants of adjuvant treatment choice among patients with stage IIIB/IIIC/IIID melanoma**


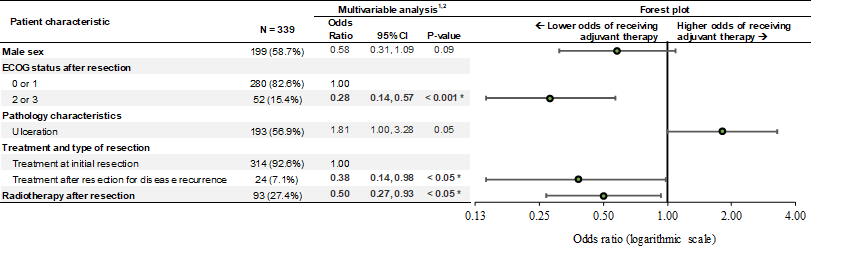

Supplement: Supplementary file 1 — Additional file 1: Supplemental Figure 1. Study design for retrospective chart review. Supplemental Figure 2. Year of surgical resection – by adjuvant therapy. Supplemental Figure 3. Determinants of adjuvant treatment choice among patients with stage IIIA melanoma. Abbreviations: CI, confidence interval; ECOG, Eastern Cooperative Oncology Group; LDH, lactate dehydrogenase. Supplemental Figure 4. Determinants of adjuvant treatment choice among patients with stage IIIB/IIIC/IIID melanoma. Abbreviations: CI, confidence interval; ECOG, Eastern Cooperative Oncology Group. [file 12885_2024_12178_MOESM1_ESM.docx]
